# Therapeutic Mechanisms of Medicine Food Homology Plants in Alzheimer’s Disease: Insights from Network Pharmacology, Machine Learning, and Molecular Docking

**DOI:** 10.3390/ijms26052121

**Published:** 2025-02-27

**Authors:** Shuran Wen, Ye Han, You Li, Dongling Zhan

**Affiliations:** 1 College of Food Science and Engineering, Jilin Agricultural University, 2888 Xincheng Street, Changchun 130118, China; wenshuran2023@163.com; 2 College of Plant Protection, Jilin Agricultural University, 2888 Xincheng Street, Changchun 130118, China; 18043340255@163.com; 3 College of Life Science, Jilin Agricultural University, 2888 Xincheng Street, Changchun 130118, China; 17790099278@163.com

**Keywords:** network pharmacology, machine learning, medicine food homology plants, molecular docking, Alzheimer’s disease

## Abstract

Alzheimer’s disease (AD) is a progressive neurodegenerative disorder characterized by a gradual decline in cognitive function. Currently, there are no effective treatments for this condition. Medicine food homology plants have gained increasing attention as potential natural treatments for AD because of their nutritional value and therapeutic benefits. In this work, we aimed to provide a deeper understanding of how medicine food homology plants may help alleviate or potentially treat AD by identifying key targets, pathways, and small molecule compounds from 10 medicine food homology plants that play an important role in this process. Using network pharmacology, we identified 623 common targets between AD and the compounds from the selected 10 plants, including crucial proteins such as STAT3, IL6, TNF, and IL1B. Additionally, the small molecules from the selected plants were grouped into four clusters using hierarchical clustering. The ConPlex algorithm was then applied to predict the binding capabilities of these small molecules to the key protein targets. Cluster 3 showed superior predicted binding capabilities to STAT3, TNF, and IL1B, which was further validated by molecular docking. Scaffold analysis of small molecules in Cluster 3 revealed that those with a steroid-like core—comprising three fused six-membered rings and one five-membered ring with a carbon–carbon double bond—exhibited better predicted binding affinities and were potential triple-target inhibitors. Among them, MOL005439, MOL000953, and MOL005438 were identified as the top-performing compounds. This study highlights the potential of medicine food homology plants as a source of active compounds that could be developed into new drugs for AD treatment. However, further pharmacokinetic studies are essential to assess their efficacy and minimize side effects.

## 1. Introduction

Alzheimer’s disease (AD) is a progressive neurodegenerative disorder characterized by cognitive decline, memory loss, and behavioral changes that significantly reduce patients’ quality of life [1,2]. With the global increase in the aging population, AD has become a leading cause of dementia, accounting for 60–80% of dementia cases [3]. The pathophysiology of AD is highly complex and involves the accumulation of amyloid plaques, tau protein tangles, neuroinflammation, and oxidative stress, collectively causing neuronal damage and loss [4]. Current treatment options are limited by significant side effects and poor efficacy, highlighting the urgent need for more effective therapeutic strategies [2].

Medicine food homology plants are defined as substances that possess both nutritional and medicinal qualities [5]. Many traditional medicines around the world include various foods, which has led to the emergence of the concept of medicine food homology [6]. These plants have long been acknowledged for their potential to promote health and alleviate various diseases [7,8]. In addition to their nutritional value, they offer therapeutic benefits that have attracted increasing interest from researchers. Their bioactive compounds, which target multiple critical proteins and pathways involved in AD progression, are promising therapeutic agents for AD treatment. In this study, we selected 10 medicine food homology plants from the list of substances classified as both food and traditional Chinese medicine (TCM) materials, as published by the National Health Commission of China, that have proven potential for treating Alzheimer’s disease (AD) and explored their anti-AD mechanisms. These plants include *Angelicae sinensis* Radix (Danggui), *Poria cocos* (Schw.) Wolf. (Fuling), *Glycyrrhizae radix* et rhizoma (licorice), *Lycii fructus* (goji berry), *Hedysarum multijugum* Maxim. (Huangqi), *Ganoderma* (Lingzhi), *Crataegi folium* (Shanzha), *Cornus officinalis* Sieb. et Zucc. (Shanzhuyu), *Ziziphi spinosae* Semen (Suanzaoren), *Gastrodiae rhizoma* (Tianma), and *Panacis quinquefolii* Radix (American ginseng) [8,9,10,11,12,13,14,15,16,17].

Given the widespread application of network pharmacology, machine learning, and molecular docking in exploring the disease mechanisms of natural plant components [18,19,20], we used these methods to investigate the potential therapeutic mechanisms of medicine food homology plants in treating AD. Network pharmacology was used to comprehensively analyze the mechanisms of action of medicine food homology plants in biological systems, and machine learning algorithms and molecular docking were used to predict the binding affinities and interactions between proteins and small molecules. This study reveals the anti-AD mechanisms and bioactive compounds of medicine food homology plants and provides a theoretical foundation and reference for developing novel AD drugs, contributing to reducing side effects and improving their efficacy.

## 2. Results and Discussion

### 2.1. Identification of Common Targets Between Medicine Food Homology Plants and AD

We collected 3401 AD-related targets from the DisGeNET [21], GeneCards [22], and PharmGKB [23] databases. Based on previous studies, 10 medicine food homology plants with potential therapeutic effects on AD were selected, including *Angelicae sinensis* Radix (Danggui), *Poria cocos* (Schw.) Wolf. (Fuling), *Glycyrrhizae radix* et rhizoma (licorice), *Lycii fructus* (goji berry), *Hedysarum multijugum* Maxim. (Huangqi), *Ganoderma* (Lingzhi), *Crataegi folium* (Shanzha), *Cornus officinalis* Sieb. et Zucc. (Shanzhuyu), *Ziziphi spinosae* Semen (Suanzaoren), *Gastrodiae rhizoma* (Tianma), and *Panacis quinquefolii* Radix (American ginseng) [8,9,10,11,12,13,14,15,16,17]. Additionally, 1507 associated targets were collected from the TCMSP [24] and TCM-ID databases [25]. To investigate the potential role of these plants in AD treatment, we performed a Venn diagram analysis to explore the overlap between AD-related and plant-related targets, as shown in Figure S1. We identified 623 common targets between AD and the plants, suggesting that these shared targets may serve as potential therapeutic targets for medicine food homology plants in AD treatment. Detailed information is provided in Table S1.

### 2.2. Protein–Protein Interaction (PPI) Network Analysis on Common Targets of AD and Medicine Food Homology Plants

**Figure 1:**
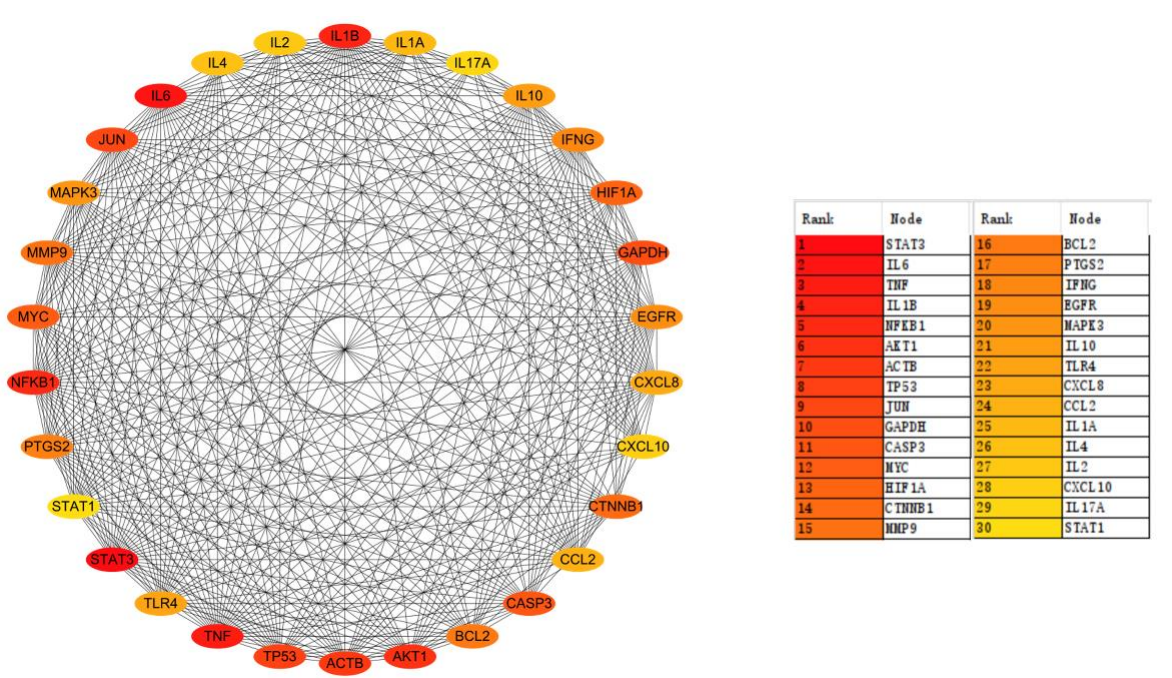
Ranking and interactions of the top 30 targets.

To explore the associations between the common targets of AD and medicine food homology plants, 623 common targets were entered into the STRING database to construct a PPI network [26]. The PPI network was visualized using Cytoscape [27], and topological analysis was performed using the MCC method with the cytoHubba plugin (v0.1), resulting in the identification of the top 30 crucial targets. The interactions and scores of these targets are shown in Figure 1. Among them, signal transducer and activator of transcription 3 (STAT3), interleukin-6 (IL6), tumor necrosis factor (TNF), and interleukin-1 beta (IL1B) were ranked as the top four targets, suggesting that they may play critical roles in AD treatment using medicine food homology plants.

### 2.3. Gene Ontology (GO) and Kyoto Encyclopedia of Genes and Genomes (KEGG) Enrichment Analysis

GO enrichment analysis is used to assess the enrichment of GO terms in a gene set. It is used to classify genes into three primary categories, namely cellular components (CC), biological processes (BP), and molecular functions (MF). The KEGG pathway enrichment analysis involves describing the roles of genes in metabolic and signaling pathways, providing insights into their functions and regulatory mechanisms.

**Figure 2:**
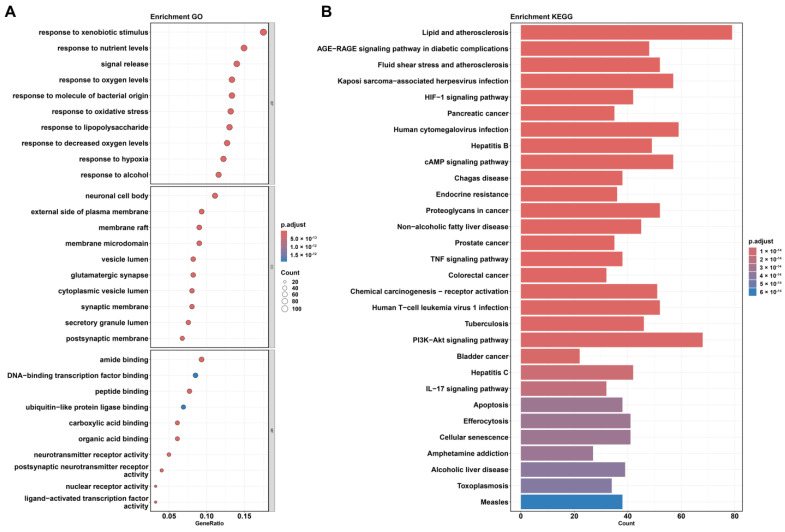
Results of (**A**) gene ontology (GO) and (**B**) Kyoto Encyclopedia of Genes and Genomes (KEGG) pathway enrichment analysis based on the common genes between Alzheimer’s disease (AD) and medicine food homology plants.

In this study, GO and KEGG enrichment analyses were performed on the 623 common targets between medicine food homology plants and AD. The results, presented as a bubble chart and bar plot, are shown in Figure 2.

Based on the BP enrichment results, medicine food homology plants may exert protective effects against AD through various biological processes.

First, “response to xenobiotic stimulus” suggests that plant compounds might influence how the body reacts to drugs, helping to regulate drug-induced side effects [28]. Enrichment in “response to nutrient levels” and “response to alcohol” indicates that these plants may improve energy metabolism and nutrient supply, potentially alleviating AD-associated metabolic abnormalities [29,30]. “Response to oxygen levels”, “response to hypoxia”, “response to decreased oxygen levels”, and “response to oxidative stress” imply that plant components might improve brain hypoxia conditions and enhance neuronal adaptability to low oxygen levels; they may also protect neurons from damage by mitigating oxidative stress [31]. “Response to lipopolysaccharide” and “response to molecules of bacterial origin” suggest that plants might reduce immune responses and suppress inflammation caused by bacterial infections, thereby slowing the progression of AD [32]. Finally, “signal release” indicates that medicine food homology plants may regulate neurotransmitter release, improving synaptic function and enhancing cognitive abilities [33].

Based on the CC enrichment results, medicine food homology plants may target various cellular structures to regulate neurofunctions associated with AD.

Firstly, the enrichment of “neuronal cell body” suggests that plant compounds may target the neuronal cell body, indicating their potential role in maintaining its health [34]. Several membrane-related enrichments, such as “external side of plasma membrane,” “membrane raft”, “membrane microdomain”, “synaptic membrane”, and “postsynaptic membrane”, indicate that plant compounds might enhance signal transmission and information exchange between neurons by regulating the structure of the cell membrane and synaptic functions [33]. Enrichment in “vesicle lumen” and “cytoplasmic vesicle lumen” suggests that plant compounds may participate in intracellular vesicular transport. Additionally, the enrichment of “glutamatergic synapse” further indicates that medicine food homology plants may improve the function of glutamatergic synapses, facilitating neural signal transmission [35]. Finally, the enrichment of “secretory granule lumen” suggests that plant compounds may regulate the function of secretory granules.

Based on the MF enrichment results, medicine food homology plants may exert protective effects on AD by regulating gene expression, protein metabolism, and neural signal transmission.

Enrichments in “amide binding”, “DNA-binding transcription factor binding”, “nuclear receptor activity”, and “ligand-activated transcription factor activity” suggest that plant compounds may regulate gene expression by interacting with transcription factors, thereby influencing AD-related cellular functions [36]. The enrichment of “peptide binding” and “ubiquitin-like protein ligase binding” indicates that plant compounds may help maintain intracellular protein homeostasis by regulating protein synthesis and degradation, potentially preventing pathological aggregation of proteins like β-amyloid [37]. Enrichment in “carboxylic acid binding” and “organic acid binding” suggests that these plants may regulate cellular metabolism by interacting with organic acids. Lastly, enrichment in “neurotransmitter receptor activity” and “postsynaptic neurotransmitter receptor activity” implies that plant compounds may enhance neurotransmitter receptor activity, improve synaptic function, and facilitate neural signal transmission [33,35].

As shown in Figure 2B, several signaling pathways were enriched in the KEGG analysis, revealing that medicine food homology plants may influence AD through multiple mechanisms.

Firstly, the “AGE-RAGE signaling pathway in diabetic complications” was notably enriched. This pathway plays a critical role in diabetes and its complications, which are known risk factors for AD. The activation of the AGE-RAGE signaling pathway exasperates neurodegenerative changes in AD, particularly in the accumulation of β-amyloid and the enhancement of neuroinflammation. Therefore, modulating this pathway could offer a novel strategy for AD treatment [38].

The “HIF-1 signaling pathway” plays a key role in cellular responses to hypoxic conditions. Studies have shown that brain regions in AD patients may suffer from insufficient oxygen supply, potentially activating the HIF-1 signaling pathway. By improving cerebral blood flow and oxygen supply, the HIF-1 pathway may help reduce hypoxic damage to neurons, thereby slowing disease progression [39].

The “cAMP signaling pathway” is also closely associated with AD. This pathway plays an important role in synaptic plasticity, memory formation, and maintenance in neurons. Disorder of the cAMP signaling pathway is commonly observed in AD patients and is linked to cognitive impairment. Restoring or enhancing the function of this pathway may improve cognitive abilities and slow cognitive decline in AD [40].

The enrichment of the “TNF signaling pathway” highlights the significant role of neuroinflammation in AD. Research has shown that inhibiting the TNF pathway reduces neuroinflammation and slows AD progression. Thus, targeting TNF signaling could be an effective therapeutic approach for AD [41].

The “PI3K-Akt signaling pathway” is involved in regulating cell survival, metabolism, and oxidative stress response. In AD, dysregulation of the PI3K-Akt pathway can lead to neuronal death and brain tissue damage. Modulating this pathway may improve neuroprotection and delay the progression of neurodegenerative changes, offering a potential therapeutic impact for AD [42].

The KEGG analysis indicates that medicine food homology plants may exert potential therapeutic effects on AD by modulating signaling pathways such as AGE-RAGE, HIF-1, cAMP, TNF, and PI3K-Akt. Interventions targeting these pathways could improve neuronal survival, reduce neuroinflammation, enhance brain metabolism and blood flow, and provide new avenues for AD treatment.

### 2.4. Cluster Analysis of Medicine Food Homology Plants

**Figure 3:**
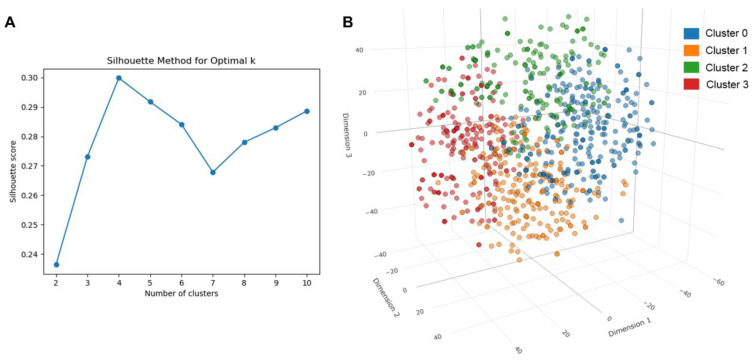
Hierarchical clustering analysis of small molecules from medicine food homology plants. (**A**) Silhouette coefficient plot used to determine the optimal number of clusters. A higher silhouette coefficient indicates better-defined and more-cohesive clustering groups. (**B**) Clustering results of 1142 small molecules based on their structural scaffolds.

To further investigate the crucial components of medicine food homology plants in AD treatment, we collected 1142 small molecular compounds from 10 food medicine homology plants using the TCMSP and TCM-ID databases. Hierarchical clustering analysis was performed based on the structural scaffolds of these small molecules, and the optimal number of clusters was determined to be four using the silhouette coefficient method. The clustering results and silhouette coefficient plot are shown in Figure 3. The SMILES of 1142 small molecules and their classification information are presented in Table S2.

### 2.5. Prediction of Binding Capabilities Between Small Molecules and Targets

**Figure 4:**
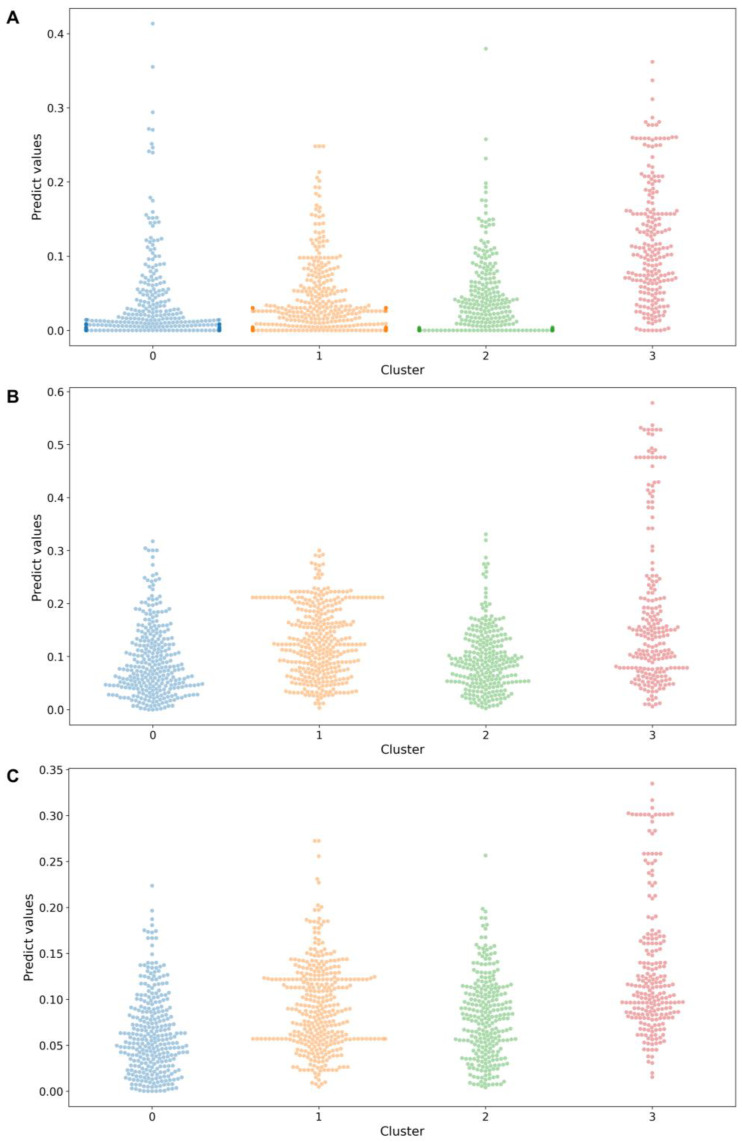
Predicted binding scores of the four clusters with (**A**) STAT3, (**B**) TNF, and (**C**) IL1B.

Among the top four crucial targets, we focused on investigating the binding capabilities of small molecules with STAT3, TNF, and IL1B because the most currently identified IL6 inhibitors are antibodies that exhibit more complex binding mechanisms than small molecules. The ConPlex algorithm was used to predict the binding capabilities between the small molecules and the targets STAT3, TNF, and IL1B. ConPlex is a sequence-based prediction model for drug–target interactions [43]. The predicted scores range from 0 to 1, with higher scores indicating stronger binding between the small molecules and the targets. The predicted scores are listed in Table S2. A classification scatter plot was used to visually present the prediction results (Figure 4), in which the binding predicted scores between each small molecule and target were quantified and represented as individual data points. As shown in the figure, Cluster 3 exhibited stronger predicted binding scores with the three targets than the other clusters, with a greater number of small molecules falling within the high-value region (above the average).

**Figure 5:**
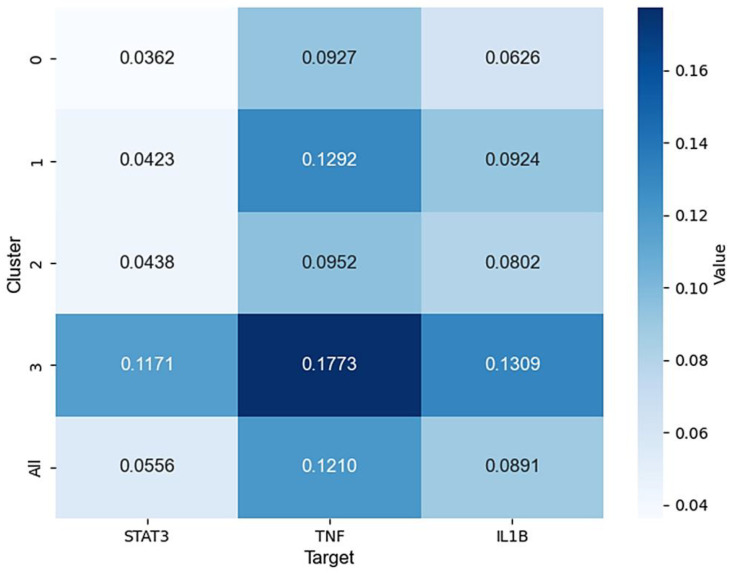
Heatmap of the average predicted binding score.

As shown in Figure 5, the heatmap displays the average predicted binding scores of the four clusters with the three targets. For STAT3, only Cluster 3 exhibited an average predicted binding score that surpassed the overall average binding score of the total small molecules. However, for TNF and IL1B, Clusters 1 and 3 showed average binding scores that were higher than the overall average. Based on these results, in the subsequent molecular docking analysis, only the representative small molecules from Cluster 3 were selected for STAT3, whereas for TNF and IL1B, representative small molecules from both Clusters 1 and 3 were chosen for docking analysis.

### 2.6. Molecular Docking of Cluster 1 and 3 with Crucial Targets

We further investigated how the small molecules in Cluster 1 and 3 interacted with the crucial targets. For each target, we selected the small molecule whose predict binding score was closest to the average within a cluster to represent the overall interaction of the entire cluster with the target. Similarly, we selected the molecule with the highest binding value within a cluster to explore its potential for more potent interactions with the target.

Table 1 presents the predicted binding affinity results for the best docking conformations, which show a trend consistent with the binding scores predicted using ConPlex (v0.1.12). The small molecules in Cluster 3 exhibited stronger predicted binding affinities for TNF and IL1B than those in Cluster 1. Among the three targets, they were predicted to bind most tightly to TNF, followed by IL1B, with the weakest binding observed for STAT3. The molecular docking results further validated the accuracy of the binding scores predicted by the ConPlex algorithm.

**Figure 6:**
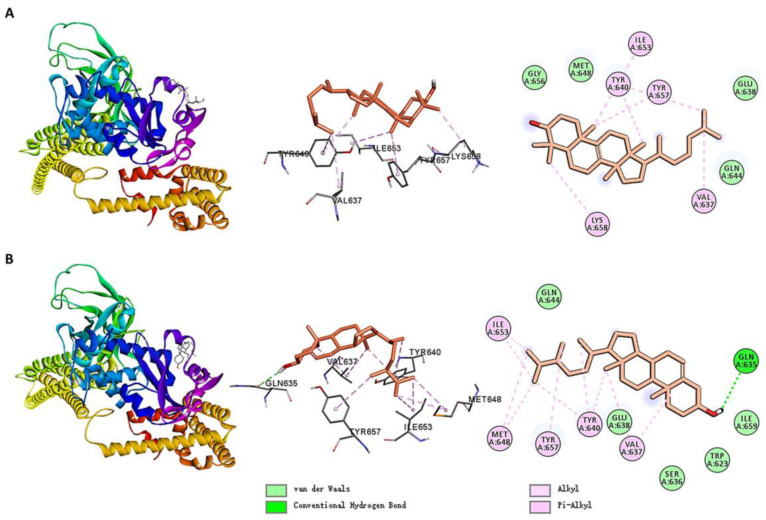
Molecular docking interactions of STAT3 with (**A**) MOL009678 and (**B**) MOL005438.

Figure 6 shows the molecular docking interactions between STAT3 and small molecules. MOL009678 formed alkyl/pi–alkyl interactions with VAL637, TYR640, ILE653, TYR657, and LYS658. Additionally, the molecule interacted with GLN644, GLU638, and other residues via Van der Waals contacts. Similarly, MOL005438 formed alkyl/pi–alkyl interactions with VAL637, TYR640, MET648, ILE653, and TYR657 while also interacting with GLN644, GLU638, and other residues via Van der Waals contacts. Notably, in contrast to MOL009678, MOL005438 formed a hydrogen bond with GLN635.

The small molecules primarily bound to STAT3 via hydrogen bonds, alkyl/pi–alkyl interactions, and Van der Waals contacts. Residues, such as GLN635, VAL637, TYR640, ILE653, and TYR657, played critical roles in the binding of the small molecules.

**Figure 7:**
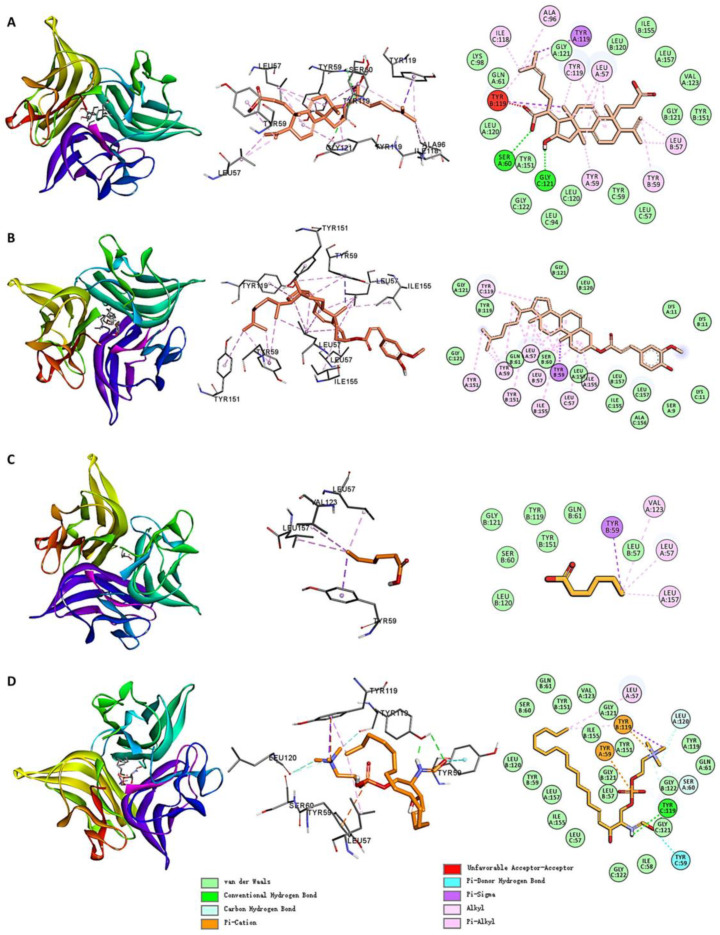
Molecular docking interactions of TNF with (**A**) MOL000291, (**B**) MOL002442, (**C**) MOL002046, and (**D**) MOL008253.

Figure 7 shows the molecular docking interactions between TNF and the small molecules. Among the small molecules in Cluster 3, MOL000291 formed alkyl/pi–alkyl interactions with residues LEU57, TYR59, and others; pi–sigma interactions with TYR119; hydrogen bonds with SER60 and GLY121; and Van der Waals interactions with LEU120, GLY121, and other residues. MOL002442 formed alkyl/pi–alkyl interactions with residues LEU57, TYR151, and others; pi–sigma interactions with TYR59; and Van der Waals interactions with LEU120, GLY121, and other residues.

Among the small molecules in Cluster 1, MOL002046 formed alkyl/pi–alkyl interactions with LEU57, VAL123, and LEU157; pi–sigma interactions with TYR59; and Van der Waals interactions with LEU120, GLY121, and others. MOL008253 formed alkyl/pi–alkyl interactions with LEU57 and TYR119 (chain B); pi–cation interactions with TYR59 and TYR119 (chain B); pi–sigma interactions with TYR119 (chain B); hydrogen bonds with TYR119 (chain C); and Van der Waals interactions with LEU120, GLY121, and other residues.

The small molecules primarily bound to TNF via hydrogen bonds, alkyl/pi–alkyl interactions, pi–sigma interactions, and Van der Waals contacts. Residues such as LEU57, TYR59, TYR119, LEU120, and GLY121 played critical roles in the binding of these small molecules.

**Figure 8:**
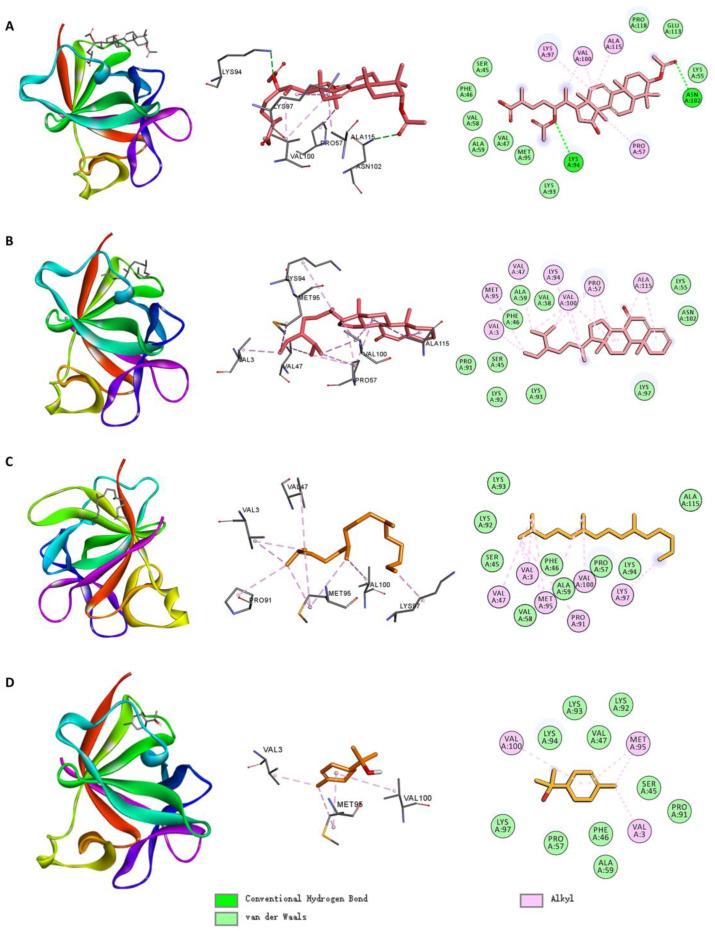
Molecular docking interactions of IL1B with (**A**) MOL011210, (**B**) MOL011442, (**C**) MOL005529, and (**D**) MOL000232.

Figure 8 shows the molecular docking interactions between IL1B and the small molecules. Among the small molecules in Cluster 3, MOL011210 formed hydrogen bonds with residues LYS94 and ASN102; alkyl interactions with residues PRO57, LYS97, VAL100, and ALA115; and Van der Waals interactions with SER45, LYS55, and other residues. MOL011442 formed alkyl interactions with residues VAL3, VAL47, PRO57, LYS94, MET95, and ALA115; and Van der Waals interactions with SER45, LYS55, and other residues.

Among the small molecules in Cluster 1, MOL005529 formed alkyl interactions with VAL3, VAL47, PRO91, MET95, LYS97, and VAL100; and Van der Waals interactions with SER45, PHE46, and other residues. MOL000232 formed alkyl interactions with VAL3, MET95, and VAL100; and Van der Waals interactions with SER45, PHE46, and other residues.

Small molecules primarily bound to IL1B via alkyl interactions and Van der Waals contacts. Residues, such as MET95, VAL100, and SER45, played crucial roles in the binding of these small molecules.

### 2.7. Identification of Potential Triple-Target Inhibitors and Analysis of Molecular Scaffolds

**Figure 9:**
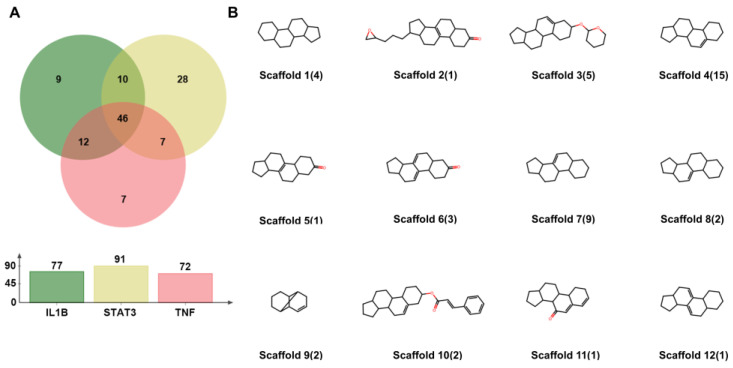
(**A**) Venn diagram showing the overlap of small molecules with predicted binding scores above the mean value for IL1B, TNF, and STAT3. (**B**) Statistical analysis of the scaffolds of the 46 small molecules, with the corresponding number of molecules for each scaffold indicated in brackets. The oxygen atom is represented in red.

Compared with the other clusters, a higher number of small molecules in Cluster 3 had binding scores that fell within the high-value region for the three crucial targets (Figure 4). Therefore, we hypothesized that some molecules in Cluster 3 would exhibit strong binding affinities for the three crucial targets. To further explore this, we identified small molecules in Cluster 3 with predicted binding scores above the mean value for each target (Figure 9A). By analyzing the overlap using a Venn diagram, we identified 46 small molecules common to the three targets. Detailed information can be found in Table S3.

Subsequently, we performed a statistical analysis of their molecular scaffolds. As shown in Figure 9B, Scaffold 4, which is present in 15 small molecules, was the most common. It comprised three fused six-membered rings and one five-membered ring with a carbon–carbon double bond, forming a steroid-like core. Scaffold 7 was the second most prevalent, sharing a similar structure with Scaffold 4 but differing in the position of the double bond, with nine small molecules containing this scaffold. Scaffold 3 was the third most common, appearing in five small molecules. It features an additional six-membered ring (containing oxygen) connected to Scaffold 4 via an ether bond.

### 2.8. Molecular Docking of Potential Triple-Target Inhibitors

We further analyzed the total predicted binding values of the 46 small molecules to STAT3, TNF, and IL1B. Molecular docking was performed on the top three molecules to investigate their binding modes with the targets. The predicted values and docking-derived binding affinities are listed in Table 2.

The predicted values of the ConPlex algorithm exhibited a trend similar to that of the docking results. MOL005439, MOL000953, and MOL005438 are potential triple-target inhibitors and promising drug candidates for AD treatment.

**Figure 10:**
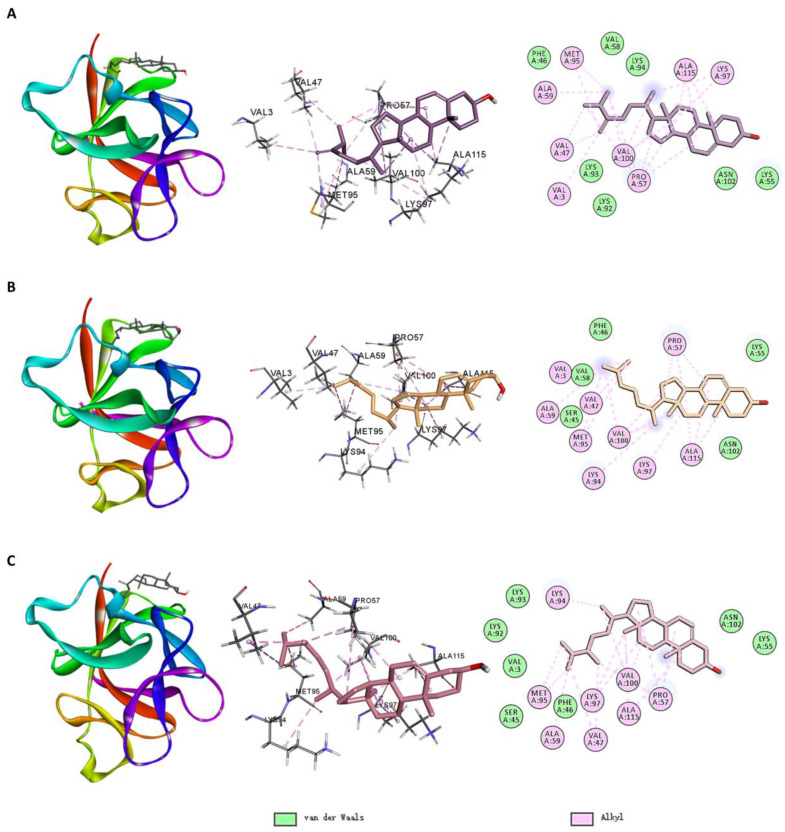
Molecular docking interactions of IL1B with (**A**) MOL005439, (**B**) MOL000953, and (**C**) MOL005438.

**Figure 11:**
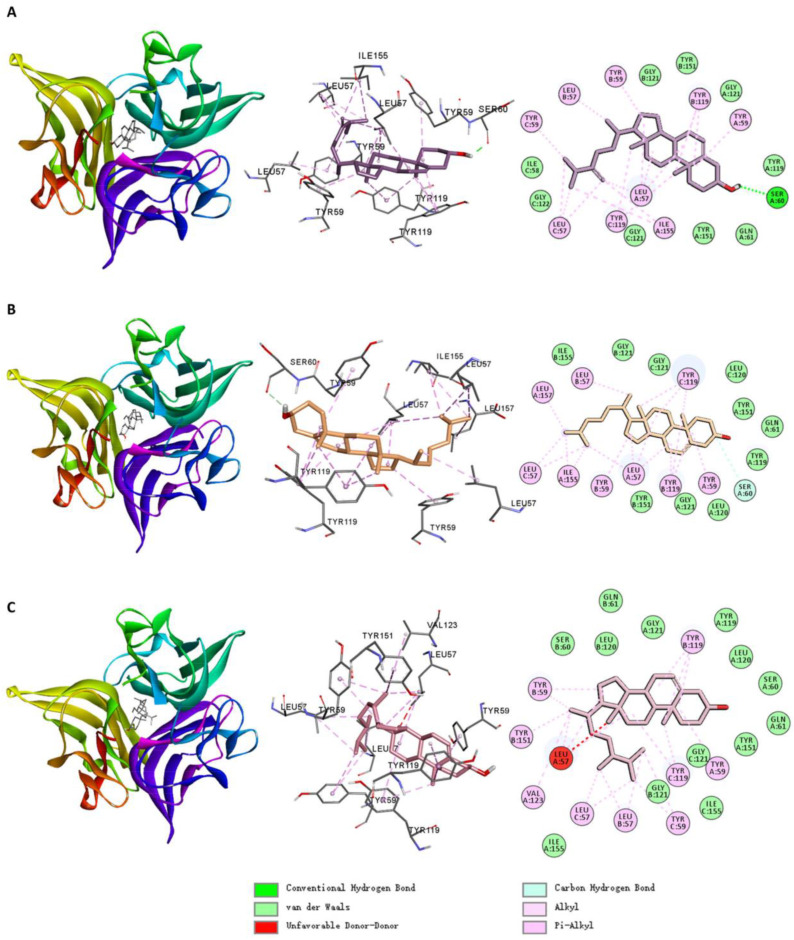
Molecular docking interactions of TNF with (**A**) MOL005439, (**B**) MOL000953, and (**C**) MOL005438.

**Figure 12:**
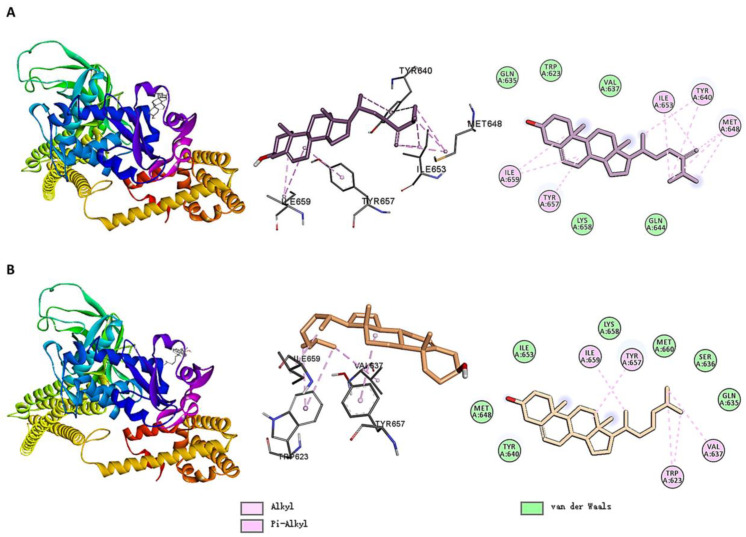
Molecular docking interactions of STAT3 with (**A**) MOL005439 and (**B**) MOL000953. The docking results of MOL005438 with STAT3 are shown in Figure 6B.

The small molecules primarily bound to the targets through alkyl/pi–alkyl interactions and Van der Waals contacts. Figure 10, Figure 11 and Figure 12 illustrate the interactions of MOL005439, MOL000953, and MOL005438 with the three target proteins.

As shown in Figure 10, for IL1B, MOL005439, MOL000953 and MOL005438 exhibited similar interaction patterns, forming alkyl interactions with VAL47, PRO57, ALA59, MET95, LYS97, VAL100, and ALA115; Van der Waals interactions with PHE46, LYS55, and other surrounding residues.

As shown in Figure S2, compared to the interaction between the IL1B inhibitor T9C (PDB: 8C3U) and IL1B, the three small molecules share the following similarities: MOL005439 and T9C both form alkyl interactions with VAL3, VAL47, PRO57, ALA59, MET95, LYS97, and VAL100, while MOL005438 and MOL000953, like T9C, form alkyl interactions with VAL47, PRO57, ALA59, MET95, LYS97, and VAL100. Additionally, MOL005439 and T9C both form van der Waals interactions with PHE46, LYS55, VAL58, and LYS92; MOL000953 and T9C both form van der Waals interactions with SER45, PHE46, LYS55, and VAL58; and MOL005438, like T9C, forms van der Waals interactions with SER45, PHE46, LYS55, and LYS92. All residues involved in the interaction between MOL005439, MOL000953, MOL005438, and target IL1B are included within those of T9C.

However, there are notable differences: T9C forms five hydrogen bonds with GLU50, LYS93, MET95, LYS97, and ASN102 and also exhibits pi–sigma and pi–cation interactions with some residues of IL1B, which are not seen with the three small molecules.

As shown in Figure 11, for TNF, MOL005439, MOL000953, and MOL005438 formed alkyl/pi–alkyl interactions with LEU57, TYR59, TYR119, ILE155, and other residues and Van der Waals interactions with ILE58, GLY121, and nearby residues. Notably, MOL000953 formed carbon–hydrogen bonds with SER60, whereas MOL005439 formed conventional hydrogen bonds with the same residue. This hydrogen bond may explain the higher calculated predicted binding affinity of MOL005439 to TNF.

As shown in Figure S3, compared to the interaction between the TNF inhibitor UTM (PDB: 6X82) and TNF, the three small molecules share the following similarities: MOL005439, MOL000953, and UTM all form alkyl interactions with LEU57 and ILE155, while MOL005438 and UTM both form alkyl interactions with LEU57. MOL005439 and UTM both form van der Waals interactions with GLN61, TYR119, GLY121, GLY122, and TYR151, while MOL000953, MOL005438, and UTM all form van der Waals interactions with GLN61, TYR119, LEU120, GLY121, and TYR155. Additionally, MOL000953 and UTM both form carbon–hydrogen bonds with SER60.

However, there are notable differences: UTM formed three hydrogen bonds with TYR151 (chain A), TYR151 (chain B), and TYR191 (chain C), and also exhibited pi–pi and pi–sigma interactions with other residues, which were absent in the interactions of the three small molecules with TNF. Furthermore, ILE58 (observed in MOL005439-TNF interactions) and VAL123 (identified in MOL005438-TNF interactions) were absent in the corresponding interaction profile of UTM.

As shown in Figure 12, for STAT3, the three small molecules formed alkyl/pi–alkyl interactions with different residues. MOL005439 interacted with ILE653, TYR640, MET648, TYR557, and ILE659; MOL000953 interacted with TRP623, VAL637, TYR657, and ILE659; and MOL005438 formed alkyl/pi–alkyl interactions with VAL637, TYR640, MET648, ILE653, and TYR657. Additionally, the three molecules exhibited Van der Waals interactions with surrounding residues. Notably, MOL005438 formed a hydrogen bond with GLN635, which may have contributed to its higher calculated predicted binding affinity for STAT3.

As shown in Figure S4, compared to the interaction between the STAT3 inhibitor KQS (PDB: 6NJS) and TNF, the three small molecules shared the following similarities in their interactions with TNF: MOL005439 and KQS both formed van der Waals interactions with LYS658, TRP623, and GLN635; MOL000953 and KQS both formed van der Waals interactions with TYR640, GLN635, MET660, and LYS658; and MOL005438 and KQS both formed van der Waals interactions with TRP623 and ILE659.

However, there are notable differences: KQS primarily bound to target STAT3 through hydrogen bonds, forming a total of 11 hydrogen bonds with residues such as SER611 and GLU612, which were absent in the interactions of the three small molecules with STAT3. Additionally, in the interaction of MOL005439, MOL000953, and MOL005438 with STAT3, MET648 and ILE653 were not included in the corresponding interaction profile of KQS.

In summary, by examining the interactions of the positive inhibitor with the three targets and comparing them to the interactions of the three small molecules, we found that, for IL1B and TNF, the small molecules exhibit similar alkyl/pi–alkyl and van der Waals interactions with the positive inhibitor. Although the modes of interaction are not exactly the same, the residues involved in these interactions overlap significantly with those of the positive inhibitor. Given this, further structural modifications based on these small molecules could potentially lead to effective inhibitors of IL1B and TNF.

For STAT3, since the positive inhibitor primarily interacts with STAT3 through hydrogen bonds and attractive charges, the three small molecules show only partial similarity in their van der Waals interactions with the positive inhibitor. The relatively weaker binding of the small molecules to STAT3 is consistent with their lower positive affinity in docking studies. Optimizing these small molecules to function as STAT3 inhibitors may require more substantial structural modifications.

Furthermore, when compared to the positive inhibitors of IL1B, TNF, and STAT3, the three small molecules share a common drawback. Specifically, in the binding of the positive inhibitor to IL1B, TNF, and STAT3, five, three, and eleven hydrogen bonds were observed, respectively. Hydrogen bonding is a crucial drug–target interaction that is lacking in the three small molecules. Therefore, further structural modifications could focus on improving hydrogen bond formation. Additionally, further pharmacokinetic studies are needed to evaluate the efficacy of these small molecules.

### 2.9. ADMET Prediction of MOL005439, MOL000953, and MOL005438

In drug development, other important factors such as transport, blood–brain barrier (BBB) crossing, and toxicity must also be considered. We performed an ADMET evaluation of the three small molecules, and the results are shown in Table S4.

Compared to the marketed drug Donepezil, the three small molecules exhibited poorer Caco-2 permeability, suggesting that they may have lower in vivo drug permeability. Further modifications to these small molecules could be made to improve this limitation.

For oral bioavailability, although the three small molecules did not perform as well as Donepezil, they were predicted to maintain at least 20% oral bioavailability.

The BBB parameter indicated that all three small molecules demonstrated good blood–brain barrier crossing ability, outperforming Donepezil. Additionally, existing research suggests that natural compounds may reduce the risk of AD or delay its onset through the gut–brain axis [44].

Toxicity predictions showed that MOL005438 has similar hepatic and renal toxicity to Donepezil, while MOL005439 and MOL000953 were predicted to have lower hepatic and renal toxicity than Donepezil. Furthermore, the predicted neurotoxicity for all three small molecules was lower than that of Donepezil. Notably, our choice of medicine food homology plant compounds for this study was also driven by their general suitability for direct consumption and typically lower toxicity.

In conclusion, compared to the existing drug Donepezil, the three small molecules of medicine food homology plants exhibited superior blood–brain barrier penetration and lower neurotoxicity. Among them, MOL005439 and MOL000953 also showed lower predicted hepatic and renal toxicity. Although their Caco-2 permeability and oral bioavailability (≥20%) were weaker than that of Donepezil, absorption can potentially be optimized through structural modifications or novel drug formulations. These small molecules thus have promising potential for further research and development as AD treatments.

## 3. Materials and Methods

### 3.1. Identification of AD-Related Targets

First, potential AD gene targets were screened from the DisGeNET (https://www.disgenet.org/, accessed on 30 October 2023), GeneCards (https://www.genecards.org/, accessed on 28 October 2023), and PharmGKB (https://www.pharmgkb.org/, accessed on 30 October 2023) databases. We identified 3397 potential targets from the DisGeNET database, 659 with relevance scores > 20 from GeneCards, and 21 from PharmGKB. After performing a union operation on the potential targets from these three databases and filtering for human proteins using Swiss-Prot (https://www.uniprot.org/, accessed on 30 November 2023), 3401 unique potential targets were identified.

### 3.2. Identification of Medicine Food Homology Plant Targets and Components

The targets and compounds of 10 medicine food homology plants were obtained from the TCMSP database (https://old.tcmsp-e.com/tcmsp.php, accessed on 16 September 2024). For *Gastrodia elata*, which was not available in TCMSP, we supplemented the information using the TCM-ID database (https://bidd.group/TCMID/, accessed on 16 September 2024). After removing duplicates from both the targets and small-molecule components, a total of 1507 unique targets and 1142 distinct compounds were obtained.

### 3.3. Using Venn Diagram to Identify Common Targets and PPI Network Analysis

We used the online tool jvenn (https://jvenn.toulouse.inra.fr/app/example.html, accessed on 17 September 2024) [45] to assess the overlap of AD targets and medicine food homology plants. The PPI network was constructed based on data sourced from the STRING database (http://string-db.org/, accessed on 17 September 2024) to evaluate the potential interactions among the identified targets. We selected all active interaction sources, and the connection weights were set to medium confidence (0.4). A visual representation of these interactions was obtained using Cytoscape 3.9.1. Subsequently, using the cytoHubba plugin, we analyzed the topological properties of the network using the MCC method and identified the top 30 targets.

### 3.4. GO and KEGG Enrichment Analysis

First, we used the “org.Hs.eg.db” package (v3.8.10) to convert the target gene symbols into standard Entrez gene IDs, ensuring the consistency and accuracy of gene identifiers in the GO and KEGG enrichment analyses. Subsequently, we performed GO and KEGG enrichment analyses using the “enrichGO” and “enrichKEGG” functions, respectively, from the “clusterProfiler” package (v4.11.0). For GO analysis, we covered the three major ontologies (BP, MF, and CC) and selected significantly enriched terms with *p*- and q-values < 0.01. Similarly, for KEGG pathway analysis, we selected significant pathways with *p*- and q-values < 0.01. The results were visualized using dot and bar plots to provide a more intuitive representation of the enrichment findings.

### 3.5. Cluster Analysis of the Components of Medicine Food Homology Plants

To cluster the 1142 small molecules based on their scaffolds, we first obtained the SMILES strings for each molecule and used the RDKit library to extract their Murcko scaffolds [46]. Subsequently, we computed second-order Morgan fingerprints (2048 bits) to represent the scaffold structures. To reduce the dimensionality of the fingerprints, we used t-SNE to map them into a three-dimensional space, generating 3D coordinates for each molecule. This approach was used to effectively group structurally similar molecules.

The silhouette method was used to determine the optimal number of clusters. We calculated the silhouette scores for clustering solutions ranging from 2 to 10 clusters and identified the most appropriate number. The clustering solution with the highest silhouette score, corresponding to k = 4, was selected. Finally, we applied agglomerative hierarchical clustering to the reduced dimensional data and grouped the molecules into categories. Each category represents a set of molecules with similar Murcko scaffolding characteristics.

### 3.6. ConPlex Prediction of Affinity Between Small Molecules and Targets

In this study, the ConPlex algorithm was used to predict the binding capabilities between crucial targets and small molecules. ConPlex integrates pre-trained protein language models with contrastive learning algorithms [47]. It predicts the binding capabilities of small molecules to target proteins based on the chemical structure of the molecules, provided in SMILES string format, and the amino acid sequences of the target proteins. We configured ConPlex locally and applied it to predict the binding capabilities of 1142 small molecules to key targets.

### 3.7. Molecular Docking

We searched the UniProt database for the gene targets STAT3, TNF, and IL1B and identified their corresponding human protein UniProt entries as P40763, P01375, and P01584 [48]. Using these entries, we then searched the RCSB PDB database (https://www.rcsb.org/, accessed on 2 December 2024) for protein–ligand complex structures, selecting 6NJS, 6X82, and 8C3U as the protein structures for molecular docking. Small molecules were obtained from TCMSP in MOL2 format. The docking sites were determined based on the positions of the positive inhibitors in the PDB complex structures.

For receptor preparation, Discovery Studio 2021 was used to remove water molecules, and AutoDock was used to add hydrogen and charges. In the 8C3U structure, which contains two identical IL1B protein–ligand complexes, we retained only one protein structure for docking. Regarding 6X82, in which the small-molecule ligand was located at the center of the three TNF proteins, the three TNF proteins were retained.

For ligand preparation, AutoDock was used to add hydrogen and charges. Molecular docking was subsequently performed using AutoDock Vina 1.2.0 [49], and the docking results were visualized using Discovery Studio 2021.

### 3.8. Analysis of Molecular Scaffolds

Jvenn was used to analyze the overlap of small molecules in Cluster 3, as obtained using the method described in Section 3.5. The intersection was based on small molecules whose predicted binding scores were above the mean for the three targets.

Subsequently, using the RDKit library, we extracted the Murcko scaffolds of the potential triple-target inhibitors in Cluster 3, counted the frequency of different scaffolds, and visualized the small molecule scaffolds using RDKit’s drawing module.

### 3.9. ADMET Prediction

To predict the absorption, distribution, metabolism, excretion, and toxicity of small molecules, we utilized the ADMET evaluation module of ADMETLAB 3.0 (https://admetlab3.scbdd.com/, accessed on 13 February 2025) [50] by submitting the SMILES formulas of MOL005439, MOL000953, and MOL005438.

## 4. Conclusions

In this study, we used an integrated computational approach to investigate how medicine food homology plants may alleviate or potentially treat AD. By combining target identification with PPI network analysis, we identified 623 common targets shared between AD and the compounds from the selected 10 plants. These targets are likely to play critical roles in the therapeutic effects of medicine food homology plants on AD. Among them, STAT3, IL6, IL1B, and TNF were identified as the top four key targets through PPI analysis.

GO and KEGG enrichment analyses further revealed the involvement of these targets in biological processes and pathways that are strongly associated with the pathogenesis of AD, including inflammation, oxidative stress, and neuronal apoptosis. These findings provide a comprehensive understanding of the molecular mechanisms underlying the therapeutic effects of medicine food homology plants in AD.

Additionally, hierarchical clustering analysis of 1142 small molecules from the 10 selected plants identified four distinct clusters. Small molecules in Cluster 3 exhibited superior binding capabilities for STAT3, IL6, and IL1B, as predicted by the ConPlex algorithm, which was further validated through molecular docking simulations. Scaffold analysis revealed that potential triple-target inhibitors in Cluster 3 shared a common structure characterized by three fused six-membered rings and one five-membered ring with a carbon–carbon double bond, forming a steroid-like core.

Among the small molecules in Cluster 3, MOL005439, MOL000953, and MOL005438 were identified as the top performers, exhibiting the highest predicted binding affinities for STAT3, IL6, and IL1B. Additionally, their interaction residues share similarities with those of known positive inhibitors. ADMET predictions show that these compounds exhibit liver and kidney toxicity profiles similar to or better than Donepezil, along with favorable blood–brain barrier crossing ability. These findings suggest that these three compounds could serve as promising lead candidates for the development of multi-target therapeutics for AD.

This study highlights the potential of medicinal food homology plant compounds as a promising avenue for AD therapy, emphasizing their multitarget therapeutic potential. Future experimental validation and structural modifications of these small molecules could accelerate the development of novel AD treatments based on medicine food homology plants.

## Figures and Tables

**Table 1:** Molecular docking affinities of small molecules in different clusters with crucial targets.

ID	Cluster	Predicted Value in Cluster	Target	Predict Affinity (kcal/mol)
MOL009678	3	average	STAT3	−5.6
MOL005438	3	Best	STAT3	−5.9
MOL000291	3	average	TNF	−8.7
MOL002442	3	Best	TNF	−11.1
MOL002046	1	average	TNF	−5.3
MOL008253	1	Best	TNF	−7.8
MOL011210	3	average	IL1B	−6.6
MOL011442	3	Best	IL1B	−7.4
MOL005529	1	average	IL1B	−5.0
MOL000232	1	Best	IL1B	−5.6

**Table 2:** Predicted scores and docking-derived affinities for the top 3 small molecules.

ID	IL1B Predicted Score	TNF Predicted Score	STAT3 Predicted Score	Total Predicted Score	IL1B Predicted Affinity	TNF Predicted Affinity	STAT3 Predicted Affinity	Mean Predicted Affinity
MOL005439	0.3083	0.5206	0.2770	1.1060	−7.5	−12.6	−5.7	−8.6
MOL000953	0.3024	0.5190	0.2809	1.1023	−7.0	−12.0	−5.6	−8.2
MOL005438	0.2803	0.4590	0.3620	1.1013	−7.2	−11.0	−5.9	−8.0

## Data Availability

Data are contained within the article and Supplementary Materials.

## Supplementary Materials

The following supporting information can be downloaded at: https://www.mdpi.com/article/10.3390/ijms26052121/s1.
