# Supplementary material for: Therapeutic Mechanisms of Medicine Food Homology Plants in Alzheimer’s Disease: Insights from Network Pharmacology, Machine Learning, and Molecular Docking
Source: Int J Mol Sci. 2025 Feb 27;26(5):2121. doi: 10.3390/ijms26052121 (PMC11899993; doi:10.3390/ijms26052121)
Supplement: Supplementary file 1 [file ijms-26-02121-s001.zip › Supplementary Figures.pdf]

Supplementary Figures

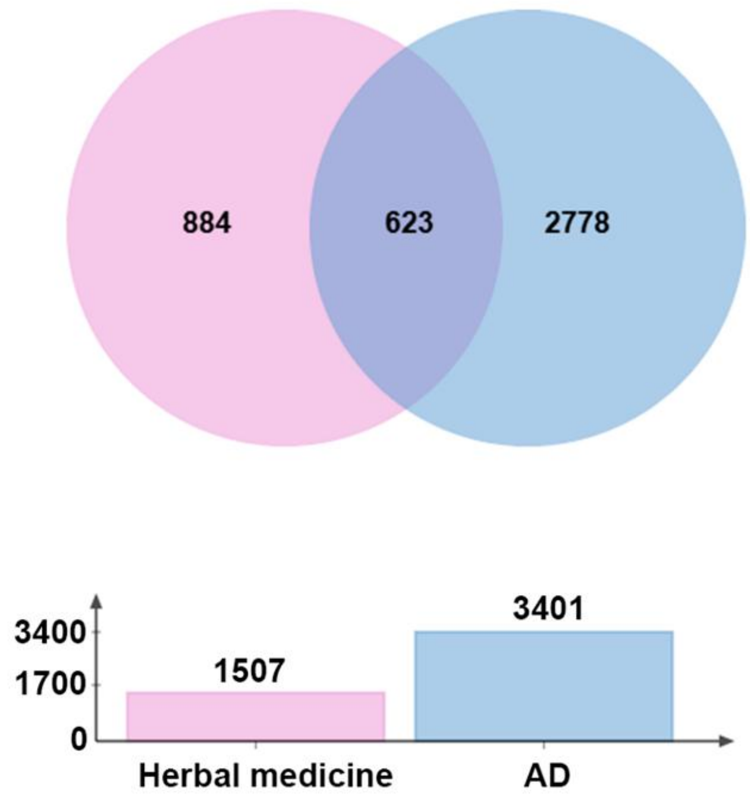

Figure S1. Venn diagram showing the 623 common targets between Alzheimer's disease (AD) and the 10 medicine food homology plants

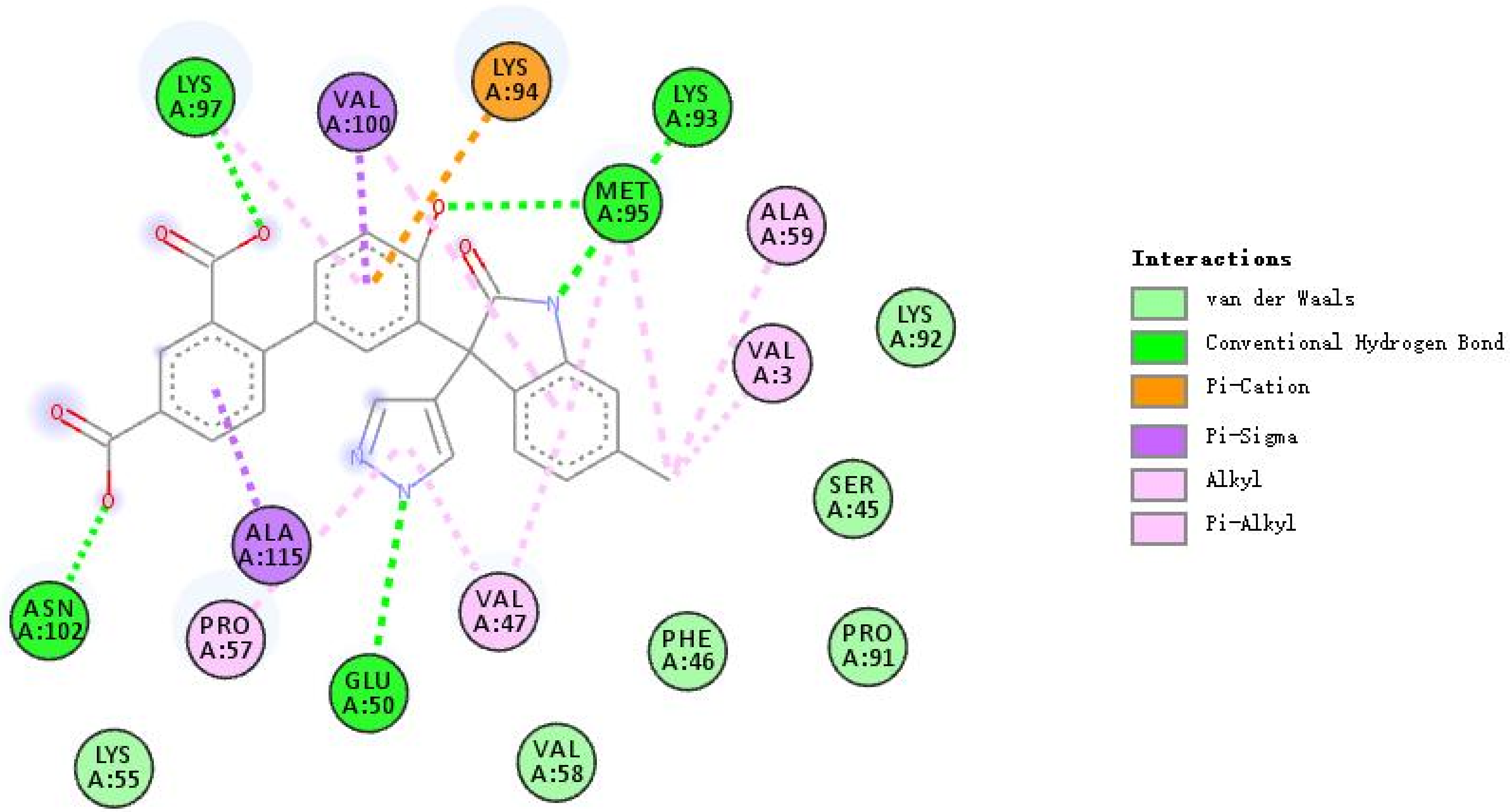

Figure S2. Molecular docking interaction of IL1B with T9C

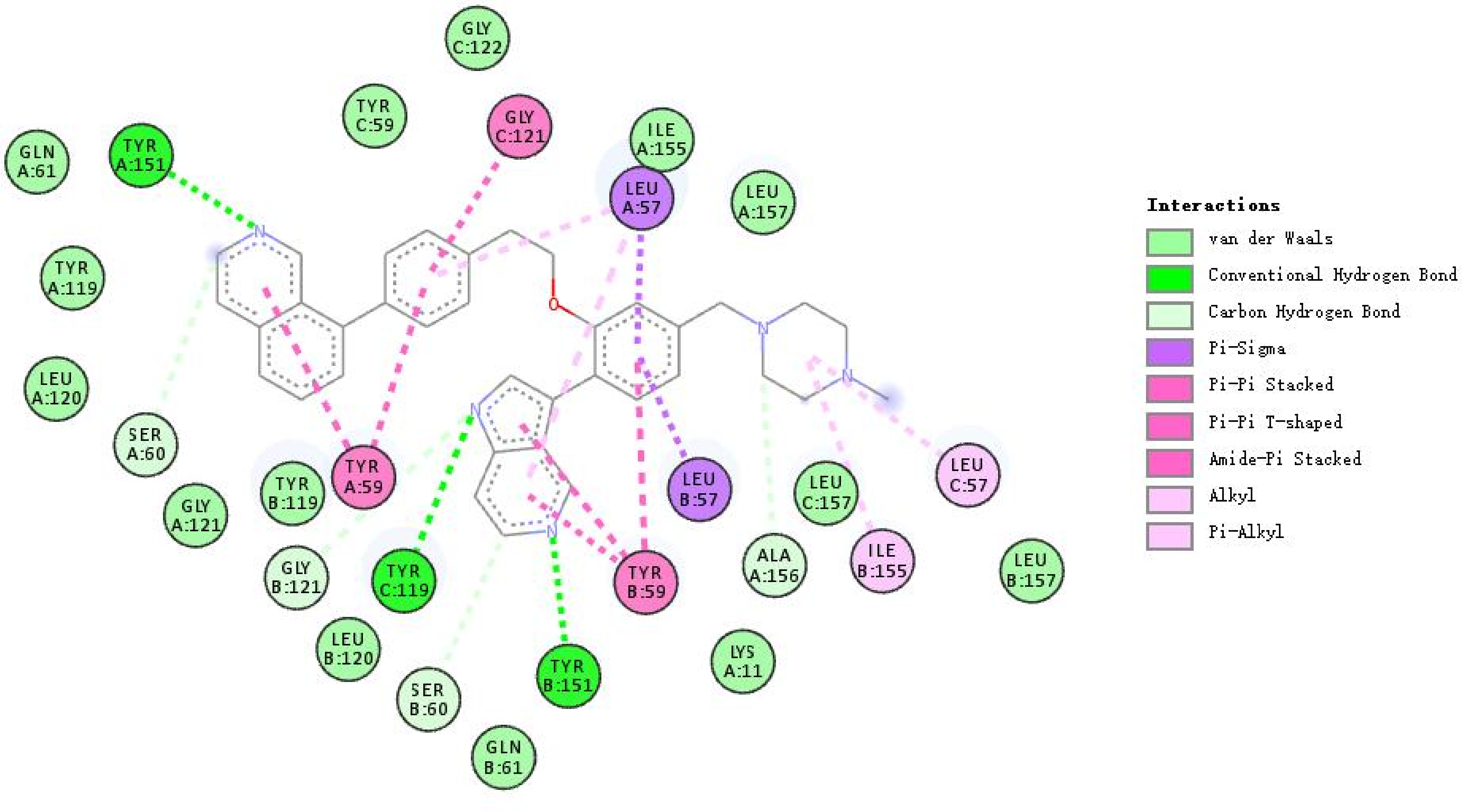

Figure S3. Molecular docking interaction of TNF with UTM

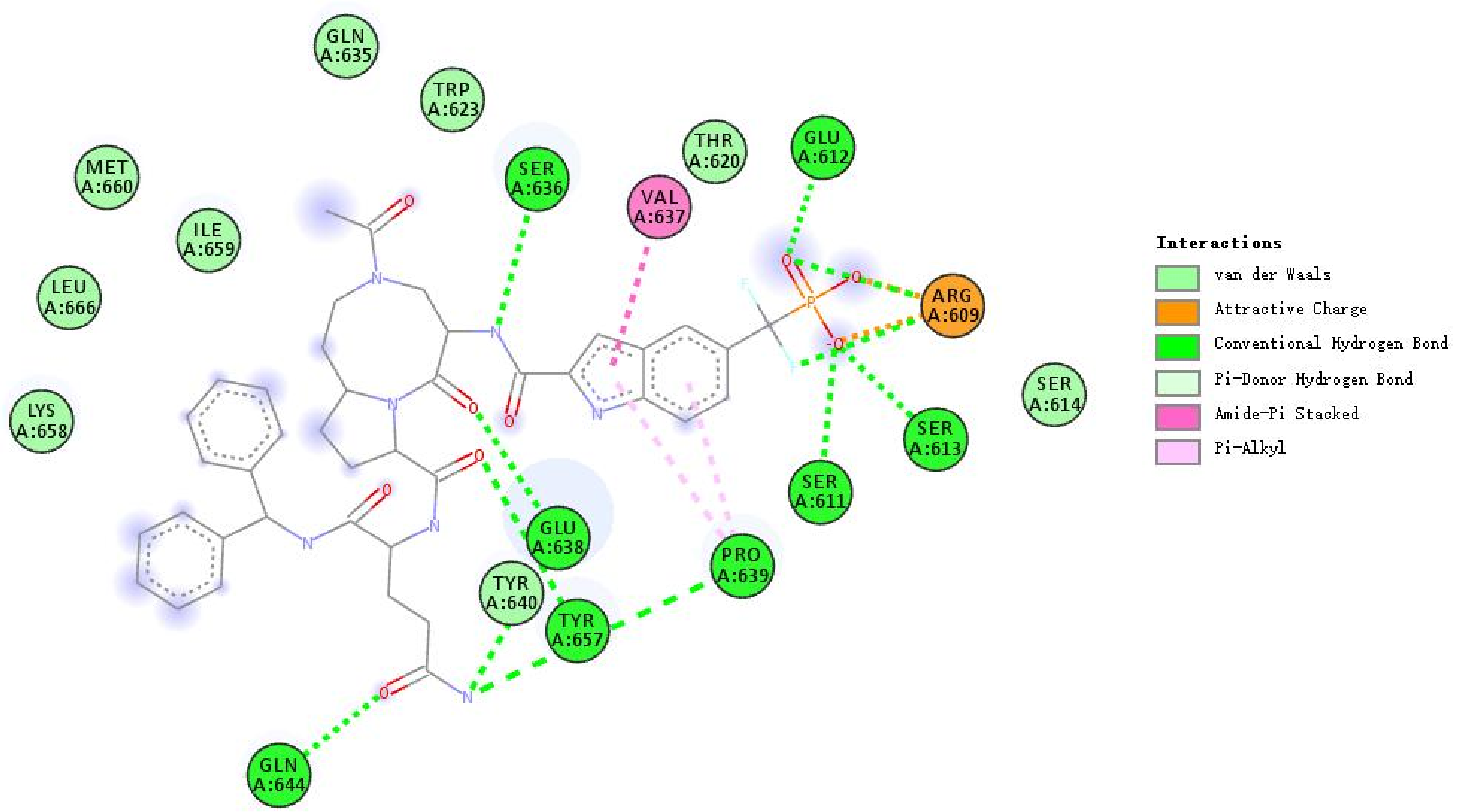

Figure S4. Molecular docking interaction of STAT3 with KQS
